# Distribution and Ecological Risk of *Ludwigia peploides* in South Korea

**DOI:** 10.3390/biology13100768

**Published:** 2024-09-27

**Authors:** Aram Jo, Soo In Lee, Donghui Choi, Youngha Kim, Yong Ho Lee, Sun Hee Hong

**Affiliations:** 1 Invasive Alien Species Team, National Institute of Ecology, Seocheon 33657, Republic of Korea; 2 Department of Biological Science, Kongju National University, Kongju 32588, Republic of Korea; 3 National Ecosystem Survey Team, National Institute of Ecology, Seocheon 33657, Republic of Korea; 4 School of Applied Science in Natural Resources & Environment, Hankyong National University, Anseong 17579, Republic of Korea

**Keywords:** *Ludwigia peploides*, invasive alien plants, ecological risk, field survey

## Abstract

**Simple Summary:**

The water primrose (*Ludwigia peploides*) is believed to have been planted as an ornamental aquatic plant in South Korea. It spread to natural ecosystems through rivers, and its distribution is gradually expanding in Suwon, Hwaseong, Busan, and Jeju. However, there has been no specific study on the ecological risk of *L. peploides* introduced into South Korea. This study, therefore, investigates the distribution status and ecological risks of *L. peploides* in South Korea through field surveys and allelopathic material analysis, as well as assessing abiotic risk factors. The distribution was confirmed at a total of 19 sites. The species was observed to primarily inhabit aquatic environments but were also observed to inhabit moist terrestrial areas such as riverbanks or wet grasslands. The results of this study are expected to aid in the identification of the current distribution and potential ecological risks of *L. peploides* in South Korea, providing vital information for the development of effective management strategies.

**Abstract:**

The number of alien species introduced into South Korea continues to increase over the years. In particular, several plants have been introduced as ornamentals. *Ludwigia peploides*, which is native to the Americas and Australia, is believed to have been planted as an ornamental aquatic plant called “water primrose” and “primrose”. It spread to natural ecosystems through rivers, and its distribution is gradually expanding in Suwon, Hwaseong, Busan, and Jeju. However, there has been no specific study on the ecological risk of *L. peploides* introduced into South Korea. This study, therefore, investigates the distribution status and ecological risks of *L. peploides* in South Korea through field surveys and allelopathic material analysis, as well as assessing abiotic risk factors. The distribution was confirmed at a total of 19 sites, with high-density mats of a single species forming along the water’s edge and on the water surface. The maximum distribution area was 13,922 m^2^ in Chilgok Reservoir in Anseong. Stems and plant fragments transported along waterways were continuously forming colonies through vegetative propagation. When evaluating the overall risk, it is determined that *L. peploides* has a high potential to cause significant damage to the ecosystem if not managed promptly. Therefore, continuous monitoring is necessary to effectively manage and prevent the habitat expansion of *L. peploides*. The results of this study are expected to aid in the identification of the current distribution and potential ecological risks of *L. peploides* in South Korea, providing essential data for ecological risk assessment and the development of effective management strategies.

## 1. Introduction

The disappearance of the geographical boundaries for species due to globalization, along with habitat environmental changes caused by climate change and global warming, provides opportunities for some alien species to migrate and establish themselves in new habitats. In some cases, these species, referred to as invasive alien species (IAS), negatively impact the richness and abundance of native species, potentially driving them to extinction and significantly threatening the biodiversity [1,2,3,4]. Additionally, the economic damage caused by IAS, affecting agriculture, livestock industries, infrastructure, and more, exceeds USD 1.28 trillion globally [5]. The international community has made efforts to prevent the invasion and spread of alien species. Several parties gathered and adopted the Kunming-Montreal Global Biodiversity Framework (GBF) in Montreal, Canada, in 2022. Among the 23 targets of the framework, the sixth is to “reduce the introduction of IAS by 50% and minimize their impact”. This goal is to be achieved by 2030 in 19 countries, including South Korea [6].

The number of confirmed alien species has dramatically increased in South Korea from 1109 species in 2011 to 2653 species in 2021 [7]. Among the several alien species that have entered the country, the Ministry of Environment designates those considered highly harmful to the ecosystem as “ecosystem-disturbing species” based on the Act on the Conservation and Use of Biological Diversity (hereafter, the “Biodiversity Act”). These species are prioritized for management, and their import, cultivation, sale, transportation, and release are legally prohibited [8]. Each year, the Ministry of Environment conducts an ecological risk assessment of specific alien species reported to be harmful, as per Article 21-2 of the Biodiversity Act, to determine whether they should be designated as ecosystem-disturbing species. This assessment involves a literature review on the impact of the species in invaded regions and field surveys and analyses to determine if they pose similar risks to the domestic ecosystem.

Aquatic and riparian ecosystems are highly vulnerable to the invasion of invasive alien aquatic plants (IAAPs) [9,10]. River corridors and waterways are among the most heavily invaded ecosystems globally [11]. The primary reasons for this are the heterogeneous and diverse structures of river ecosystems, high connectivity, and the ease of propagule transportation [12,13]. *Ludwigia* genus are among the most aggressive, invasive plants in the world [14,15]. One of them, *Ludwigia peploides* ((Kunth) P.H. Raven, 1963) is a perennial water plant belonging to the Onagraceae family, native to North America, South America, and Australia. It has now spread worldwide to the United States, Australia, New Zealand, France, Belgium, and Greece in southern Europe due to the increased use of *Ludwigia* species as ornamental aquatic plants [16,17]. It exhibits a great deal of morphological variation depending on habitat conditions with stems tending to be erect in shallow waters and spreading laterally underwater [14,18]. Stems that spread at the surface of the water produce new roots at the nodes, and cut stems and leaves can also form new plants through vegetative propagation. Seeds and plant parts can spread long distances along the water. High-density mats of a single species formed on the water surface can replace the habitat of existing native plants, reduce species diversity, and cause several problems such as increasing sedimentation and restricting water flow when they proliferate at reservoir entrances or waterways [14,16,17,19].

In South Korea, there is a record of this plant being studied for horticultural purposes in 2003, and it has been distributed and planted as ornamentals, such as “water primrose” and “primrose”. *L. peploides* have spread into natural ecosystems through rivers, and their distribution is gradually expanding in Suwon, Hwaseong, and Busan [20]. However, there has been no specific study on the ecological risk of *L. peploides* introduced into South Korea. This study, therefore, investigates the distribution status and ecological risks of the *L. peploides* in South Korea through field survey and allelopathic material analysis, as well as assessing abiotic risk factors such as water quality, temperature, depth, and flow rate. This is the first ecological risk analysis of *L. peploides* in South Korea, addressing biotic and abiotic invasion factors.

## 2. Materials and Methods

The investigation items for assessing the ecological risk *of L. peploides* followed the ecological risk assessment table outlined in regulation No. 239 (2023) of the National Institute of Ecology (NIE). The risks not observed in domestic populations were compiled through a review of the literature.

### 2.1. Distribution Status and Biological Risk Factors

To identify the distribution of *L. peploides*, the areas where the distribution has earlier been reported were collected from the Guide for Nationwide Survey of Non-native Species in Korea (2015–2022) and the National Ecosystem Survey (2014–2022), along with literature reviews of papers and reports documenting its distribution in South Korea. Among the reported areas, four sites with high population densities—Boryeong, Osan, Busan, and Anseong—were selected for fixed plot survey (one point in the lentic zone and three points in the lotic zone). Quadrats (1 m × 1 m, *n* = 6) were established, and vegetation surveys were conducted using the Braun-Blanquet method. The relative frequency (RF) and relative coverage (RC) of species within the quadrats were used to calculate the species diversity index (Shannon–Wiener index) and species evenness index. Differences in these indices based on the presence of *L. peploides* were tested using one-way ANOVA (R, ver. 4.3.1, San Francisco, CA, USA). A flora survey was conducted to identify species co-occurring with *L. peploides*, and the importance value (IV) was calculated using the average of RF and RC to determine the importance of each species within the populations.

Fixed survey areas were set in Boryeong, Osan, Busan, and Anseong to investigate the spread of *L. peploides*. Within these survey areas, the distribution area was measured three times. The first measurement was from May to June, the second from July to August, and the third from September to October. The distribution area observed above the water surface was determined by visual inspection and drone shooting. The distribution area calculations were performed using Quantum Geographic Information System (QGIS ver. 3.16.8). The populations were continuously observed along the 1.5 km from Gonam 2 Bridge to Yeonji Bridge in Bongdangcheon Stream in Boryeong. However, for the quantitative analysis, a certain section (110 m × 30 m) was selected as the survey area where distribution monitoring was more manageable. The survey area (380 m × 5 m) was set along the stream flowing through the area around Malgeumteo Park in Osancheon Stream in Osan. In Busan, the survey area was set along approximately 640 m of the Suyeong River flowing from Sincheon Bridge towards Hoedong Reservoir. Chilgok Reservoir in Anseong was the only lentic zone among four fixed survey areas; the entire reservoir was, therefore, set as the scope of the survey. Additionally, to assess productivity, spots with 100% coverage of *L. peploides* were selected during its growing season in July and September at Chilgok Reservoir, which had the largest distribution area among the fixed survey areas. Quadrats (1 m × 1 m, *n* = 5) were used to measure the number of flowers, fruits, and seeds per unit area (1 m^2^) at each spot.

*L. peploides* has been reported to exhibit high invasiveness, including reducing the germination rates of surrounding aquatic plants and exerting toxic effects by using allelopathic compounds, which are more abundant compared to other aquatic plants [21,22]. Allelopathic interactions may play a key role in the success of invasive plants because they can alter physiological processes and thereby influence community structure [23,24]. Secondary compounds previously researched in the *Ludwigia* genus were polyphenols, flavonoids, quercitrin, prunin, myricitrin, etc. [21,25]. Additionally, the research on selecting secondary compounds for evaluating the risk of invasive alien plants indicated that when compounds such as total phenolic compounds (TPCs), caffeic acid, and p-coumaric acid are present in high concentrations, there is a higher likelihood of negative effects on native plants [26]. In this study, therefore, six compounds were included for the analysis of allelopathic compounds: the total flavonoid content (TFC), total phenolic content (TPC), caffeic acid, p-coumaric acid, myricitrin, and prunin. Leaf and root samples for allelopathic analysis were collected in August from Boryeong, Osan, and Suwon, and the analysis was conducted at the National Instrumentation Center for Environmental Management (NICEM, Seoul, Republic of Korea). TFC and TPC analyses were performed using a spectrophotometer (Spectramax ABS Plus, Molecular Devices, San Jose, CA, USA), while the other compounds were analyzed using HPLC (high-performance liquid chromatography, Ultimate3000, Thermo Dionex, Walthamma, MA, USA). Differences in allelopathic compounds by plant part (leaves and roots) and by habitat were tested using one-way ANOVA (R, ver. 4.3.1) to understand the mechanism of allelopathy and variations in compound types and levels across different regions.

### 2.2. Abiotic Risk Factors

In the risk assessment of invasive alien species, the habitat characteristics are fundamental abiotic factors that should be investigated. For non-native aquatic plants, it is important to determine whether they grow under the same aquatic ecosystem conditions reported to be their native range or if they adapt and establish themselves in new domestic environments. Additionally, water quality analysis can provide valuable information for identifying suitable habitats for their spread and establishment. At the five sites in Osan, Boryeong, Busan, Daegu, and Anseong, 2 L water samples were collected for analysis of water quality using sterile sampling bottles in September and October after the rainy season. The samples were stored in the dark at 4 °C until analysis. Water quality analysis of samples was performed by Gyeonggi Research Institute of Environmental Sciences (GRIES, Seoul, Korea). The total organic carbon content (TOC) was measured using the high-temperature combustion oxidation method, and the total phosphorus (TP) was determined using ultraviolet–visible spectrophotometry. The chemical oxygen demand (COD) was measured using the acidified potassium permanganate method, and biochemical oxygen demand (BOD) was determined using the amount of dissolved oxygen consumed by aerobic microbial growth and respiration. Suspended solids (SSs) were measured using the gravimetric method with glass fiber filters. Additionally, a multi-parameter water quality meter (YSI-556MPS) was used to measure water temperature, pH, water depth, and flow rate at the habitats to determine how these parameters influence the distribution of *L. peploides*.

## 3. Results

### 3.1. Distribution Status

**Figure 1:**
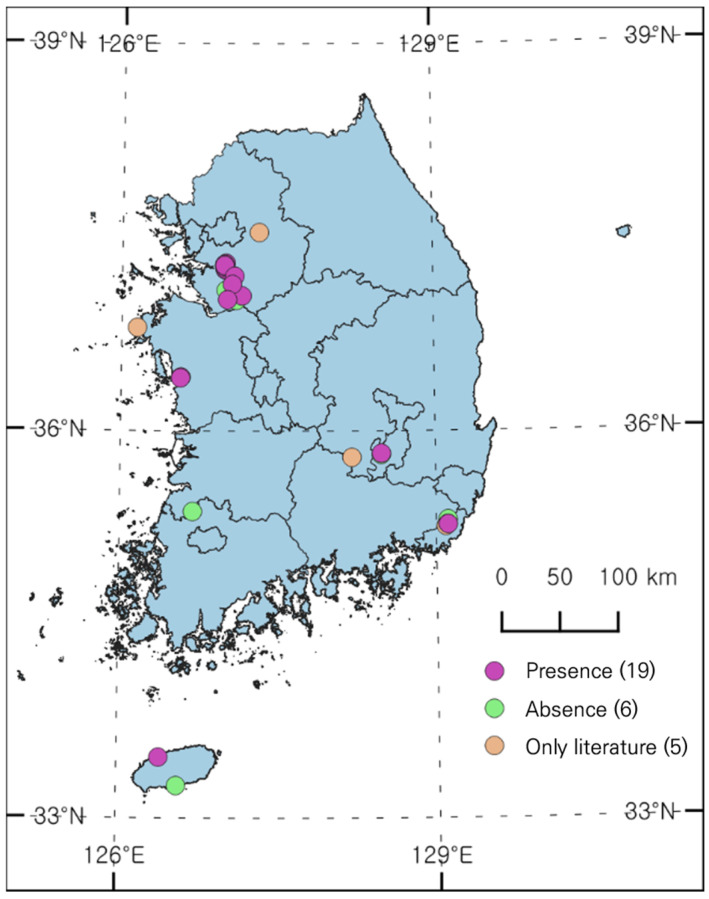
Distribution status of *L. peploides* in South Korea.

A total of 25 sites were identified with reported occurrences of *L. peploides* through literature reviews of papers and reports (Figure 1). During the initial field survey (April to early May 2023), few individuals were found at most sites except in Anseong, Osan, and Busan. However, in the second field survey (June to August), distribution was confirmed along the Seoho Stream in Suwon, from the Jangan-gu to the Paldal-gu and Gwonseon-gu, and also found in Daegu and Jeju. Only a very small number of individuals were found in Ilwang Reservoir in Suwon; a large population was previously reported, which appears to have resulted from the continuous removal of aquatic plants. Distribution was confirmed at 19 out of the 25 sites in the field survey.

### 3.2. Biological Risk Factors

**Figure 2:**
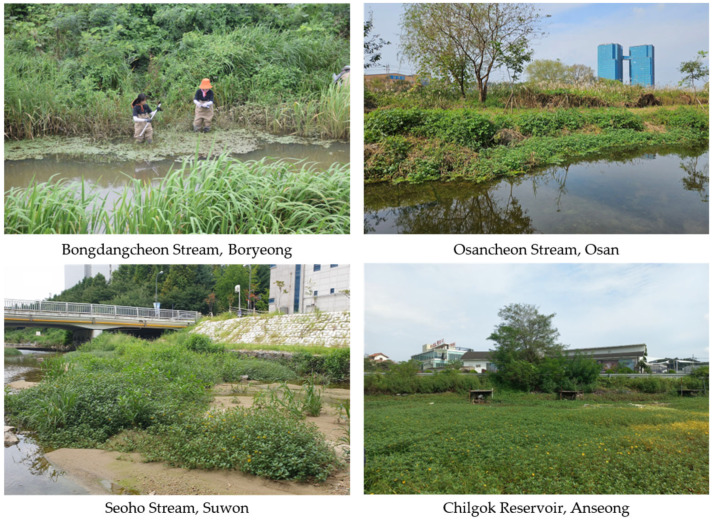
Habitats of *L. peploides* in South Korea. Boryeong: measuring the depth of the habitat of *L. peploides* in the water edge of the river. Osan: *L. peploides* inhabiting the boundary between the river and land. Suwon: large blooming populations of *L. peploides* in shallow land exposed to receding water levels under the bridge. Anseong: based on the road running horizontally in the middle, the reservoir below is entirely covered with the blooming *L. peploides* with yellow flowers.

A total of 25 *L. peploides* primarily inhabit aquatic environments but can also form populations on moist terrestrial areas such as riverbanks or wet grasslands. It is primarily found in wetlands and water–land boundaries in its native range [17]. It is relatively frost-resistant and has been reported to grow in water up to 3 m deep [27]. It has, however, been observed to inhabit waters up to 80 cm deep and is found at the water edge in rivers and reservoirs or in shallow land exposed to receding water levels in South Korea (Figure 2). As previously reported [28], domestic populations also demonstrated morphological variations, with erect stems, when growing on land and sprawling stems on the water surface.

The vegetation survey demonstrated that species evenness was significantly lower where *L. peploides* appeared (*p* < 0.0954). However, while species diversity tended to be slightly low, it did not show a significant difference (*p* < 0.123). The flora survey was conducted to identify species co-occurring with *L. peploides*. A total of 165 species of plants across 123 genera were observed, and no endangered plants were observed. However, the IV of emergent hydrophytes such as *Zizania latifolia, Phragmites australis*, *Typha orientalis*, and *Miscanthus sacchariflorus* presented a significant decrease.

**Figure 3:**
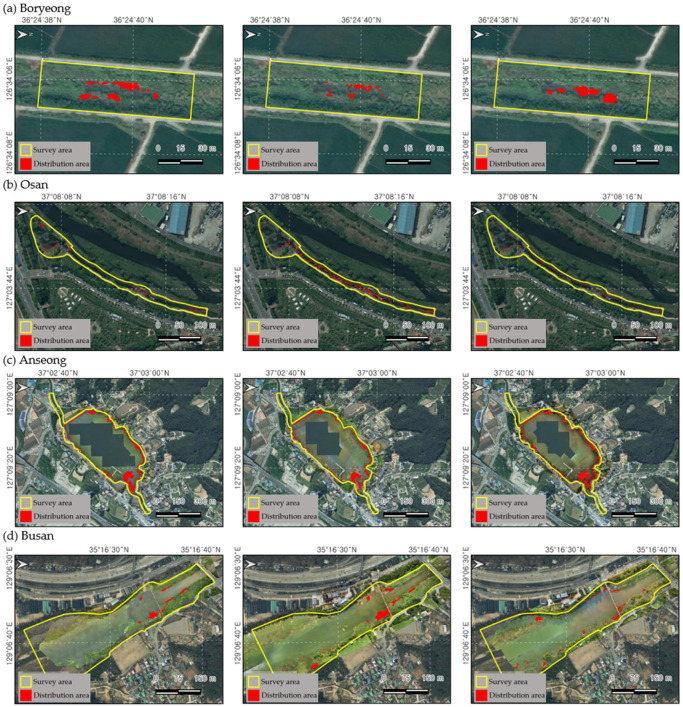
The results of the fixed plot distribution area survey to investigate the spread of *L. peploides* over time. (**a**) Bongdangcheon Stream, Byeong, (**b**) Osancheon Stream, Osan, (**c**) Chilgok Reservoir, Anseong, and (**d**) Sutyeong River, Busan. The yellow zone represents the fixed plot survey area, and the red zone shows the distribution area of *L. peploides.* From the left, the first column represents the first survey, measured from May to June; the second column represents the second survey, measured from July to August; and the third column represents the third survey, measured from September to October.

The distribution area of *L. peploides* was calculated within the fixed plot survey. In the Bongdangcheon Stream in Boryeong, the initial survey presented the largest area of 155 m^2^. However, the population was submerged below the water surface due to the higher water level caused by the summer rainy season. Further, the distribution tended to be pushed out by the competition with the co-occurring emergent plants compared to other habitats (Figure 3). However, after the rainy season, the distribution displayed dense growth on the open water surface. A similar pattern was observed in the Chilgok Reservoir of Anseong City, with an initially large area decreasing during the rainy season. In the case of Osancheon Stream in Osan and Suyeong River in Busan City, the plants presented maximum growth in August, with areas of 802 m^2^ and 2585 m^2^, respectively. However, in the Osancheon Stream, the distribution area decreased significantly due to river landscape management, and, in the Suyeong River, the distribution area decreased due to high water levels.

**Figure 4:**
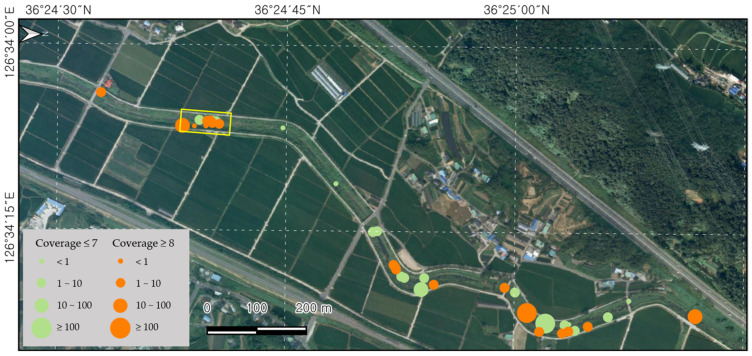
Distribution patches of *L. peploides* along the water in Bongdangcheon Stream, Boryeong. The yellow zone represents the fixed plot survey area, and the orange and circles shows the patches of *L. peploides*.

Small patches of *L. peploides* were continuously found outside the fixed plot along the water in the Bongdangcheon Stream and Suyeong River. There was a total of 38 patches in the Bongdangcheon Stream, most of which covered areas of less than 10 m^2^. However, it was found that more than half of these patches had a cover greater than eight in the Braun-Blanquet method (Figure 4). The patches were continuously observed along approximately 1.8 km from Sincheon Bridge to the entrance of Hoedong Reservoir in Suyeong River. Seed production was measured in Chilgok Reservoir in Anseong to assess productivity. In July, the number of flowers per m^2^ was 36 (±8), the number of fruits was 316 (±128), and the number of seeds was 15,561 (±7163). In September, the number of flowers per m^2^ was 15 (±2), the number of fruits was 63 (±12), and the number of seeds was 753 (±358).

The analysis of allelopathic compounds in the populations of *L. peploides* from Boryeong, Osan, and Suwon revealed high levels of TFC and TPC, with significantly more detected in the leaves than in the roots (*p* < 0.001, Table 1). This trend was consistent across all regions, although there were significant differences in content levels between regions (*p* < 0.001). The TFC detected in the leaves had an average value of 8.40 mg/g, with Boryeong showing the highest value at 9.56 mg/g. The TPC had an average value of 39.45 mg/g, with Boryeong and Suwon showing very high values exceeding 40 mg/g. Similarly, Boryeong had the highest amount of caffeic acid at 219.72 mg/kg, with more than seven times the amount detected in the leaves compared to the roots. P-coumaric acid was detected only in the roots across all regions, with an average value of 16.17 mg/kg, while prunin was detected only in the leaves across all regions, with an average value of 514.44 mg/kg.

### 3.3. Abiotic Risk Factors

Analysis of water quality at five sites—Osan, Boryeong, Busan, Daegu, and Anseong—demonstrated that most parameters were in the slightly good range (Table 2). However, the levels of TOC and TP were found to be at very poor levels (exceeding the standards of 8 and 0.5, respectively) in the Osancheon Stream. Similarly, the COD of the Chilgok Reservoir was at a very poor level (exceeding the standard of 10). The pH was in the range of pH 8 to 9 at all sites, which was more alkaline than the common range of general aquatic plants (pH 6.5 to 7.5). Furthermore, when measuring water depth as per the presence or absence of *L. peploides*, the average water depth of areas where the plant was present was 39.3 cm ± 11.9 (*n* = 15); this was lower than the water depth of areas where the plant was absent (65.3 cm ± 34.5 (*n* = 15)). During the early stages of growth, *L. peploides* was primarily observed at the edges of slow-flowing rivers and reservoirs, and, during the middle and late growth stages, it appeared to spread across the water surface.

## 4. Discussion

The genus *Ludwigia* is one of the largest and most diverse groups within the Onagraceae family. These plants are known as one of the taxa that are very difficult to identify due to their morphological similarity and wide morphological variation depending on the habitat environment [20,28]. Additionally, almost all species in the Oligospermum section, a subunit of the genus *Ludwigia*, actively hybridize, and hybrid populations adapted to their habitats can increase their invasiveness [29,30]. *L. peploides*, first identified in Suwon, is distributed in temperate and tropical regions and grows in both terrestrial and aquatic environments. As dense mats of *Ludwigia* spp. spread, they physically restrict the habitats of fish and aquatic invertebrates, and if they occur along fish migration routes, they can limit fish movement, potentially reducing biodiversity in the invaded areas [31,32]. Additionally, due to their rapid reproduction and the ability to propagate vegetative propagation from cut stems, controlling them is very challenging. Due to its harmful impact on ecosystems and agriculture, *L. peploides* is included in the European list of invasive alien species of Union concern (the Union list), and its possession, import, sale, breeding, and release into natural ecosystems are prohibited [33]. It is also included in Portugal’s national invasive species list, where its possession, breeding, and trade are prohibited [34]. In the UK, under Schedule 9 of the Wildlife and Countryside Act 1981, it is illegal to plant this species in the wild in England and Wales [35]. In New Zealand, it is classified as an “Unwanted Organism” under the Biosecurity Act 1993, and permission must be obtained for its movement, trade, and release [36]. In the wetlands of Portland, USA, labor costs for removing *L. peploides* exceeded KRW 57 million won, and physical removal costs from 2012 to 2014 amounted to approximately KRW 5 million won per acre; yet complete removal was not achieved [29,37]. 

It is understood that *L. peploides* has been introduced to South Korea as an ornamental plant and is currently observed nationwide including Suwon, Osan, Boryeong, Daegu, Anseong, and Jeju. The plant prefers slow-flowing rivers, wetlands, and stagnant reservoirs and is observed concentrated on the border between land and water surface of rivers or reservoirs. In South Korea, it is believed that a population initially planted in Ilwang Reservoir in Suwon has spread downstream to Seohocheon Strem [20]. Similarly, small patches were consistently found along the water in Boryeong and Busan. Therefore, although it is currently forming large populations in certain regions nationwide, it may continue to expand its distribution along the water. As a result of observing the change in distribution area in four fixed survey areas with high population density, it appears that July to September is the maximum growth period in South Korea. Although the rise in water levels during the rainy season influenced the growth and inflorescence of *L. peploides*, it did not significantly impact the subsequent distribution area expansion. However, the result of the habitat survey of *L. peploides* demonstrates that the population formation began with rooting on the soil at the border between land and water and spread to shallow water during the early stage of growth. It, therefore, indicates that the change in water level, affected by rain and reservoir water use, is the greatest factor in the formation of populations of *L. peploides* in South Korea.

It has been reported that *L. peploides* has tolerance to various water quality environments [21,38]. Similarly, the analysis of domestic habitats demonstrated that it actively grew even in poor water quality conditions, such as high levels of TOC and TP. It, therefore, appears that water pollution does not have a significant impact on the domestic habitat of *L. peploides*. Additionally, secondary compounds were analyzed to determine the allelopathic action of *L. peploides* on surrounding plants, and significant amounts of phenolic acids were detected, particularly in the leaves. These results are consistent with previous studies that found *L. peploides* has high invasiveness due to its higher TPC compared to other aquatic plants [22]. Phenolic compounds significantly contribute to the adaptation and growth of invasive alien plants, particularly aquatic plants, in new habitats and play a crucial role in protecting them from predation or pathogenic invasion. Invasive plants, therefore, with high phenolic compound content may have a competitive advantage over native plants [21,39]. However, further research is needed to understand the specific mechanisms by which the secondary compounds of *L. peploides* affect native plants coexisting in its habitats and the surrounding environment in South Korea.

The loss of biodiversity due to *L. peploides* was observed to be weak in this study. The results of the vegetation survey demonstrated that although the IV of co-occurring aquatic plants such as reed and bulrush decreased, there was no significant difference in species diversity. However, the high-density population mat of *L. peploides* can quickly cover the water surface, physically displacing native plant habitats and blocking light essential for submerged species. This can alter the habitat environment and potentially reduce the biodiversity of not only plants but also fish and aquatic invertebrates [14,16,21,30]. In addition, although no endangered plants were observed during the survey of flora, there have been instances in Belgium where *L. peploides* invaded protected areas with high biodiversity [19]. Since this study was conducted based on one year of observation, additional research is needed in the future to specifically, identify the risks and the impact of *L. peploides* on ecosystems in South Korea.

## 5. Conclusions

Once invaded, *L. peploides* can rapidly expand its distribution area through seed reproduction and vegetative propagation. It can survive over winter both under the water surface and below ground and repeat the generation the following year. It is, therefore, difficult to completely eradicate it. When evaluating the overall risk, it is determined that *L. peploides* has a high potential to cause significant damage to the ecosystem if not managed promptly. Therefore, continuous monitoring is necessary to effectively manage and prevent habitat expansion of *L. peploides*. Since this study was conducted based on one year of observation, additional research is needed in the future to specifically identify the risks and the impact of *L. peploides* on ecosystems in South Korea. The results of this study can help identify the current distribution and potential ecological risks of *L. peploides* in South Korea, providing essential data for ecological risk assessment. As the first nationwide survey of *L. peploides* distribution, it can be used in the future to prioritize regions for management to prevent further spread and serve as foundational data for developing effective management strategies. Additionally, this study will lay the groundwork for building risk assessment data for aquatic alien plants, aiding in the future development of more advanced and effective risk assessment methods.

## Figures and Tables

**Table 1:** The content level of allelopathic compounds between three regions (mean ± SE, *n* = 3). TFC: total flavonoid content; TPC: total phenolic content. The hyphen symbol indicates that the compound was not detected.

Region	Part	TFC (mg/g)	TPC (mg/g)	Caffeic Acid (mg/kg)	Myricitrin (mg/kg)	p-Coumaric Acid (mg/kg)	Prunin (mg/kg)
Boryeong	Leaf	9.56 ± 0.02	48.29 ± 0.68	219.72 ± 1.34	-	-	457.60 ± 3.80
	Root	2.18 ± 0.02	7.06 ± 0.11	27.65 ± 1.80	-	17.73 ± 0.24	-
Osan	Leaf	7.77 ± 0.10	27.96 ± 0.08	142.14 ± 0.42	-	-	499.81 ± 1.30
	Root	0.78 ± 0.04	0.43 ± 0.02	-	-	15.94 ± 0.06	-
Suwon	Leaf	7.88 ± 0.01	42.09 ± 0.33	147.94 ± 1.13	8681.84 ± 26.13	-	585.90 ± 2.57
	Root	1.27 ± 0.04	0.41 ± 0.01	19.58 ± 0.03	-	14.84 ± 0.25	-

**Table 2:** The results of the water quality analysis at five sites. TOC: total organic carbon, TP: total phosphorus, COD: chemical oxygen demand, BOD: biochemical oxygen demand, SS: suspended solids.

Parameter (mg/L)	Osan	Boryeong	Busan	Dague	Anseong	Average
TOC	8.3	2.7	2.2	6.1	3.9	4.64
TP	0.879	0.059	0.27	0.653	0.077	0.3876
COD	10.5	4.4	5.8	9	13.3	8.6
BOD	3.3	1.5	2.1	3	2.6	2.5
SS	5	3.2	67	27.8	9.2	22.44

## Data Availability

All data generated or analyzed during this study are included in this published article.
